# An Isotope Dilution Mass Spectrometric (IDMS) Method for the Determination of Vitamin C in Milk

**DOI:** 10.6028/jres.093.080

**Published:** 1988-06-01

**Authors:** P. Ellerbe, L. T. Sniegoski, J. M. Miller, E. White

**Affiliations:** Center for Analytical Chemistry, National Bureau of Standards, Gaithersburg, MD 20899

The accurate determination of constituents in food is necessary to establish dietary requirements. A non-fat milk powder (NFMP) has been developed as a Standard Reference Material (SRM) for use in validating methods for the analysis of milk and other biological materials. When this SRM was issued, L-ascorbic acid (vitamin C or AA) was measured by HPLC methods developed at NBS and by the AOAC microfluorimetric method.

An IDMS method has been developed as an independent method to measure AA in NFMP, using AA-13 C-l as an internal standard. After the labeled AA is added to the NFMP and has equilibrated with the unlabeled AA present, samples are treated to remove protein, put over an anion exchange column, and freeze-dried. AA is converted to the t-butyldimethylsilyl derivative and analyzed by capillary column GC/MS under a strict measurement protocol [1]. The two masses of interest are the [M-C_4_H_9_]^+^ ions at 575 and 576. The ions are monitored using a mass switching system designed to provide accurate measurement of isotope ratios for the narrow GC peaks (12–15 seconds) typical of capillary columns.

Two independent sets of standards were prepared by weight from labeled and unlabeled ascorbic acid. Each set was used as standards to measure, according to the protocol, the weight ratios of the other set as if it were samples. The difference between the measured and weighed weight ratios within each set is calculated as a percent difference, which is a measure of the consistency of each independently prepared set with the other. As an example, the average percent difference for set 1 as standards and set 2 as “samples” was +0.06%. This set is consistent with the other set because the mean of the percent differences is acceptably low.

Analyses of the NFMP Standard Reference Material run in 1984 gave values of 53 (SD = 5) µg/g by an HPLC method [2] and 49.6 (SD=4.2) by the AOAC method [2,3]. Analyses run in 1987 by two other HPLC methods [4] gave values of 35 (SD = 1) and 41.4 µg/g. Table 1 gives the results of the IDMS analyses; the IDMS overall mean is 40.23 µg/g (SD = 0.71).

There are two points to consider. Firstly, the value for AA appears to have fallen over 3 years, suggesting that AA is not stable in this matrix. It must be noted, however, that the measurement methods used in 1984 and 1987 were not the same. Secondly, there is a difference between the HPLC measurements made in the same year, and that one result does not agree with the IDMS result, thus indicating a problem with at least one of the methods.

In summary, L-ascorbic acid can be determined precisely in NFMP by an ID/MS method using a C-13 labeled ascorbic acid as the isotopic diluent. The discrepancy between the HPLC and IDMS methods is being investigated, but since the L-ascorbic acid content of this Standard Reference Material may change rapidly with time, a certified value for AA will not be assigned.

## Figures and Tables

**Table 1:** Results of ID/MS analyses

Bottle	Sample	µg AA/g NFMP	SD	CV(%)
1	1	40.31		
	2	39.85		
	mean	40.08	0.33	0.82
2	1	41.11		
	2	40.55		
	3	41.16		
	4	41.21		
	5	41.01		
	6	39.68		
	mean	40.79	0.59	1.45
3	1	39.88		
	2	39.37		
	3	39.15		
	4	39.76		
	5	39.91		
	mean	39.61	0.34	0.85
	overall mean	40.23	0.71	1.77
